# Facile Fabrication of Self-Similar Hierarchical Micro-Nano Structures for Multifunctional Surfaces via Solvent-Assisted UV-Lasering

**DOI:** 10.3390/mi11070682

**Published:** 2020-07-14

**Authors:** Shuo Zhang, Qin Jiang, Yi Xu, Chuan Fei Guo, Zhigang Wu

**Affiliations:** 1 State Key Laboratory of Digital Manufacturing Equipment and Technology, School of Mechanical Science and Engineering, Huazhong University of Science and Technology, Wuhan 430074, China; shuo_zhang@hust.edu.cn (S.Z.); jiangqin@hust.edu.cn (Q.J.); xuyi_sr@hust.edu.cn (Y.X.); 2 Department of Materials Science and Engineering, Southern University of Science and Technology, Shenzhen 518055, China; guocf@sustech.edu.cn; 3 School of Optical and Electronic Information, Huazhong University of Science and Technology, Wuhan 430074, China

**Keywords:** self-similar structures, hierarchal micro–nano structure, solvent-assisted UV-lasering, ultra-broad high light absorbance, stretchable electronics, bending sensor

## Abstract

Cross-scale self-similar hierarchical micro–nano structures in living systems often provide unique features on surfaces and serve as inspiration sources for artificial materials or devices. For instance, a highly self-similar structure often has a higher fractal dimension and, consequently, a larger active surface area; hence, it would have a super surface performance compared to its peer. However, artificial self-similar surfaces with hierarchical micro–nano structures and their application development have not yet received enough attention. Here, by introducing solvent-assisted UV-lasering, we establish an elegant approach to fabricate self-similar hierarchical micro–nano structures on silicon. The self-similar structure exhibits a super hydrophilicity, a high light absorbance (>90%) in an ultra-broad spectrum (200–2500 nm), and an extraordinarily high efficiency in heat transfer. Through further combinations with other techniques, such surfaces can be used for capillary assembling soft electronics, surface self-cleaning, and so on. Furthermore, such an approach can be transferred to other materials with minor modifications. For instance, by doping carbon in polymer matrix, a silicone surface with hierarchical micro–nano structures can be obtained. By selectively patterning such hierarchical structures, we obtained an ultra-high sensitivity bending sensor. We believe that such a fabrication technique of self-similar hierarchical micro–nano structures may encourage researchers to deeply explore the unique features of functional surfaces with such structures and to further discover their potentials in various applications in diverse directions.

## 1. Introduction

In nature, there are huge examples of hierarchical micro–nano structures with self-similar features in living systems such as lotus leaves [1], nepenthes [2,3], and geckos [4,5,6]. In particular, due to such self-similar hierarchical micro–nano structures, they often demonstrate attractive properties or unique behaviors such as super hydrophobicity [7,8,9,10,11,12] or hydrophilicity [13,14,15,16], low frication [17,18,19,20], and controllable adhesion [21,22,23,24,25], and they serve as never-ending inspiring resources for researchers to develop artificial materials and smart devices [26,27]. Hence, it is important to seek for new ways to make/replicate such special structures as simply as possible in a well-controlled manner, as well as to further exploit the corresponding potentials in diverse applications. Among these applications, from the perspective of surface engineering, a self-similar morphological surface with a high fractal dimension often has a relative larger surface area and hence demonstrates higher performance in multiple fields such as super hydrophilicity or hydrophobicity for anti-fog or self-cleaning, low friction, high (catalysis) reactivity, controlled anisotropic behaviors, and highly efficient radical particle entangling [28,29,30,31,32,33]. However, the investigation of such self-similar surface fabrication and application development has not been thoroughly conducted according to the existing literature.

Silicon is a fundamental material in the modern information society, and so silicon surface patterning techniques have been extensively studied for various purposes [34,35,36]. Furthermore, it has been found that multifarious rough micro–nano surface morphologies offer numerous opportunities to enrich functional performances, such as black Si that is capable of effective light absorbance during a wide spectral range and energy conversion for solar cells, energy harvesting in triboelectric nanogenerators, and high performance actuation in electro-wetting platforms [37,38,39,40,41].

Numerous techniques have been established to obtain micro/nanoscale structures on black Si surface to obtain a high light absorbance or low reflectivity. A general approach is to deposit a layer of an anti-reflection coating, such as SiO_x_, TiO_x_, ZnO, ITO, or Si_3_N_4_ [42]. However, this approach’s performance is severely constrained due to the fact that such coatings only work well in circumscribed spectral ranges and highly depend on incident angle [43]. An alternative approach like anisotropic etching with a solution mixture of KOH/NaOH and ethanol/isopropanol provides a rough surface of microstructural “pyramids” with more pits and spikes [43,44]. Unfortunately, such an approach is very sensitive to the crystal orientation, so its performance (reflectivity) is severely affected by incident angle due to isotropic microscale “pyramid” structures. Recently, techniques based on reactive ion etching (RIE) and metal catalyzed chemical etching (MCCE) have been developed. Nevertheless, they also have some problems, such as costly facility, required operational conditions, and wasted liquid that may be harmful to the natural environment [37,45,46,47,48,49,50,51,52,53]. An appealing technique is that of directly ablating silicon surface to obtain “penguin-like” microstructures via femtosecond lasering in toxic gas atmospheres such as SF_6_, Cl_2_, and H_2_S [51,54,55,56,57,58,59,60,61,62]. In such an approach, the performance of absorbance is not consistent in a wide spectrum, i.e., it is high in the ultraviolet spectrum but low in the infrared. Thus, exploiting a tractable surface morphology modification of black Si with a simplified fabricating process that also exhibits high performance is still attractive for society.

In this work, by introducing solvent-assisted UV-lasering in an ordinary laboratory environment, we established a facile and rapid approach to obtain self-similar hierarchical micro–nano structures for multifunctional surfaces. With the help of organic solvents, we could tune the morphologies (microgrooves) of such hierarchical structures by adjusting the operational parameters of the UV-laser. The obtained surface was found to have a high fractal dimension, a high light absorbance in an ultra-broadband spectrum (>97.5% in 200–400 nm; >94.9% in 400–750 nm; and >90% in 750–2500 nm), a high efficiency of heat transferring, and super-hydrophilicity. Furthermore, through selective replication, silicone rubber can be utilized for soft electronics self-assembling. More importantly, by doping carbon particles into a silicone substrate, hierarchical micro–nano structures can also be obtained with our solvent-assisted UV-lasering. Based on such multiscale structures on silicone rubber, we demonstrated an ultrasensitive bending sensor (ΔR/R_0_ up to 800%). Such easily obtained. artificially self-similar hierarchical structures with high fractal dimensions may trigger the further deep study of the unique features of functional surfaces at the micro–nano scale and may further develop new possibilities or applications that may benefit academia, industry research, and our daily lives.

## 2. Experimental

### 2.1. The Solvent-Assisted UV Lasering Process

In this work, N-type Si <100> wafers (CZ, 4-inch, one-side polished, with a resistivity of 0.001–50 Ω cm and a thickness of 500 µm) were purchased from Kaihua Shunchen Electronic Technology Co. LTD (QuZhou, China). A UV-laser marker (HGL-LSU3/5EI, Huagong Laser, Wuhan, China, wavelength: 355 nm; power: 5 W; mode: pulse; beam quality: M^2^ = 1.2; laser intensity: 12.69 × 10^6^ W/m^2^; focusing means: laser spots are focused on the surface of the materials) was introduced to directly treat the surface of a Si wafer in an ordinary laboratory environment. The laser beam was focused on the surface of the substrate as a circular spot with diameter of about 25 µm. During the lasering, the scanning speed (*v*, mm/s) and interval of adjacent laser scanning lines (Δ*L*, mm) were adjusted to modify the working parameters of the laser, therefore tuning the surface morphology of micro-/nano-structures, such as roughness and height difference. Meanwhile, the sample was placed in an isopropanol/ethanol solution to promote the re-deposition of the micro-/nano-particles. After ablation, the sample was cleaned with isopropanol (IPA) and deionized water. As for silicon rubber, the similar process was conducted.

### 2.2. Surface Characterization

The contact angles of the water droplets on different samples were measured with drop shape analysis equipment (DSA25, KRUSS, Hamburg, Germany). The surface texture, such as the 3D profile, surface roughness (Sa), and height difference, of the black Si were measured with an ultra-depth three-dimensional microscope (DXS 510, Olympus, Tokyo, Japan). The detailed micro surface morphology and elemental analysis of the black Si was observed with a field scanning electron microscope (FSEM, GeminiSEM300, Carl Zeiss, Jena, Germany) and a laser scanning confocal microscope (VK-X200K, Keyence, Osaka, Japan). 

### 2.3. Light Absorption and Heat Transfer Observation

The light absorption of the black Si was measured using a UV–vis–NIR spectrometer with a direct detection unit (SolidSpec-3600, Shimadzu, Kyoto, Japan) in the 200–2500 nm wavelength range. Real-time infrared temperature images of samples were taken with a thermal image camera (FLIR T630sc, FLIR Systems AB, Täby, Sweden), and samples were heated on a hot plate set at 110 °C. 

### 2.4. Self-Assembly of Soft Electronics

The soft substrate with selectively patterned hydrophobic/superhydrophobic or liquid alloy-wetted/super liquid alloy-phobic areas was fabricated following the schematic shown in Supplementary Figure S1. Firstly, the Si wafer was patterned with the UV-laser. Then, the Si wafer with patterns was cleaned with IPA, and then it was dried with a nitrogen gun. Next, the Si master was coated with a perfluoro surfactant, (1H, 1H, 2H, 2H-perfluorooctyl trichlorosilane 97%, Alfa Aesar, Shanghai, China) in a glass desiccator for at least 4 hours before use. After coating, the thoroughly stirred polydimethylsiloxane (PDMS) mixture (10:1 of weight ratio, Sylgard 184, Dow Corning Corporation, Midland, MI, USA) was poured on the Si master and cured at 75 °C for 45 min in an oven (UF 55 plus, Memmert, Schwabach, Germany). Finally, the soft PDMS substrate was peeled off with care. The LED (0805, green) was bought from Taobao (Alibaba, Hangzhou, China). The liquid alloy (Galinstan) was purchased from Geratherm Medical AG, Germany. The self-assembly process was recorded with the drop shape analysis equipment from the side view and a digital camera (Canon EOS 70D, Tokyo, Japan) from the top view.

### 2.5. Self-Cleaning Demonstration

The UV-treated Si sample (area of 30 × 40 mm^2^) was firstly treated with several droplets of 1H, 1H, 2H, 2H-perfluorooctyl trichlorosilane/ethanol solution (5 wt%) and dried in ambient air. Consequently, the superhydrophilic Si was tuned to be superhydrophobic. The self-cleaning process was recorded with the same camera above (Canon EOS 70D, Tokyo, Japan).

### 2.6. Fabrication and Characterization of Bending Sensor

We employed a cPDMS (10 g of silicone base, 1 g of curing agent, and 0.2 g of carbon black (XC72R, CABOT, Alibaba, Hangzhou, China)) of 200 µm thickness cured at 75 °C for 45 min, following UV lasering with parameters of *v* = 400 mm/s and Δ*L* = 0.05 mm to obtain the surface with self-similar hierarchical micro–nano structures. Then, the sample was coated with a ~100 nm thick Ag layer in a vacuum evaporation system (Mini-Spectros, Kurt J. Lesker) at <5 × 10^−7^ Torr. The resistance of the bending sensor was measured with a multimeter (34461A, Keysight Technologies, Shanghai, China).

## 3. Results and Discussion

### 3.1. The Process of Solvent-Assisted UV Lasering

**Figure 1:**
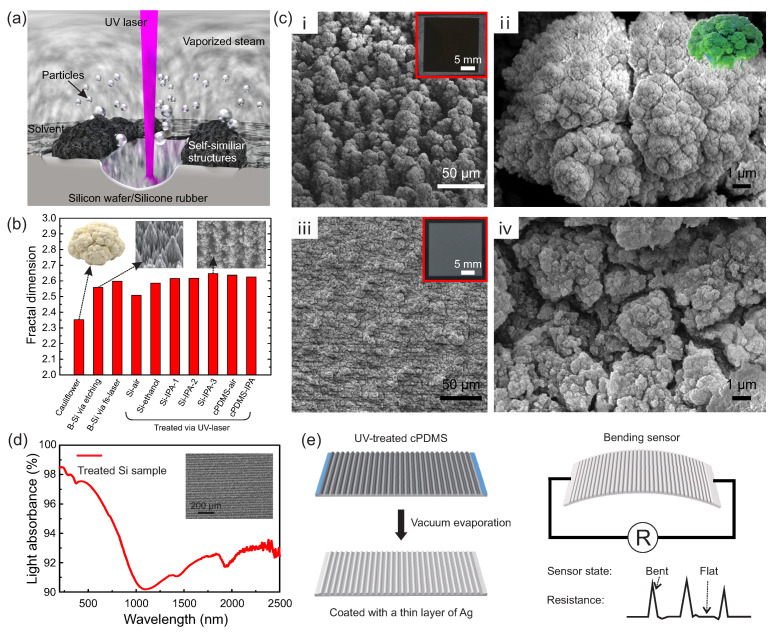
Fabrication, observation, and characterization of self-similar hierarchical micro–nano structures on diverse surfaces. (**a**) Schematic fabrication process of hierarchical structures with a solvent-assisted UV-lasering. (**b**) Micro-views of the treated Si sample with an area of 2 × 2 cm^2^ under *v* = 15 mm/s and *ΔL* = 0.02 mm in the IPA (**i**,**ii**) and cPDMS (**iii**,**iv**) samples with an area of 2 × 2 cm^2^ under *v* = 400 mm/s and *ΔL* = 0.01 mm in IPA. (**c**) Calculated fractal dimensions of several self-similar surfaces. (**d**) The light absorbance of a treated Si sample in the range of 200–2500 nm. The inset shows a magnified view of the sample. (**e**) An illustration of the UV-treated cPDMS sample coated with a 100 nm thick layer of Ag applied as a bending sensor.

As shown in Figure 1a and Figure S1, the sample was directly treated with a solid-state UV laser (355 nm), on the condition of being coated with a layer of IPA or ethanol in ambient air. The pulse repetition frequency and width were fixed as 40 kHz and 1 µs for silicon or 50 kHz and 0.1 µs for silicone rubber, respectively. We adjusted the scanning speed (*v*) and space between scanning lines (Δ*L*) to tune the surface texture of the samples. During laser ablation (Figure 1a), the pulsed laser beams ablated the substrate material (silicon wafer/silicone rubber), resulting in sputtered particles with micro-/nano-size (the arrow), vaporized steams (the “fog”), and self-similar hierarchical structures (the detailed forming mechanism is discussed in Section 3.2). After lasering, samples were cleaned with IPA and then deionized water to remove the loosely deposited mesoscopic particles. Micro-views and optical photographs of the treated Si (Figure 1i,ii) and carbon doped silicone rubber (cPDMS; Figure 1iii,iv) are demonstrated in Figure 1b. For Si (the inset in Figure 1i), the UV-treated area appeared much darker than the non-treated flat area, which indicated a significant surface topography modification and a better light absorption. Further, more detailed views of surface micro-/nano-structures (Figure 1i,iii; 1200× magnification) were investigated using a FSEM. In particular, there were extensive micro–nano structures superimposing both at “ridges” and “valleys,” which were composed of micro-peaks ranging from several to dozens of microns and nano-lumps within a few hundred nanometers. In a much closer view (Figure 1ii,iv; 25,000× magnification), a higher degree similarity could be found between our obtained structure and a truly broccoli structure than in a previous study [63].

To quantify the self-similarity of the obtained hierarchical micro–nano structures, we calculated the fractal dimensions of the diverse surfaces, including that of natural cauliflower, reported black silicon surfaces, and our obtained silicon and cPDMS surfaces (Figure 1c). The detailed calculation and programming code can be found in Supplementary Note 1, together with optical photos of samples in Figure S2. The result indicated that our UV-lasered broccoli-like structured surface exhibited higher fractal dimensions (~2.647), which led to increasing active surface and new functionalities, such as a broadband high light absorbance and anisotropic solvent absorption.

Specifically, such hierarchical structures exhibited an anisotropic forest-like texture and contained abundant small particles that helped to trap light via multiple diffuse reflections and thus improved light absorbance. When the Si sample was fabricated under the situation of *v* = 15 mm/s and Δ*L* = 0.03 mm in the IPA atmosphere, the light absorbance remained over 90% in the 200–2500 nm spectral range; see Figure 1d. This was superior to many other approaches in the cover range of the light spectrum and very useful for collecting optical energy under various natural or indoor environments without direct or strong sun shine, e.g., in overcast or cloudy days. The inset shows a micro-view of the black Si sample in which several regular micro-ridges can be observed, thus indicating a good repeatability of surface morphology via our fabrication process. For soft materials, e.g., cPDMS, surfaces with hierarchical micro–nano structures can be used to fabricate ultra-sensitive bending sensors (Figure 1e). After coating with a very thin layer of silver (~100 nm), the electrical resistance of such an anisotropic texture was extremely sensitive to bending applied on the sensor; we discuss this in detail later.

### 3.2. Mechanism Investigation

**Figure 2:**
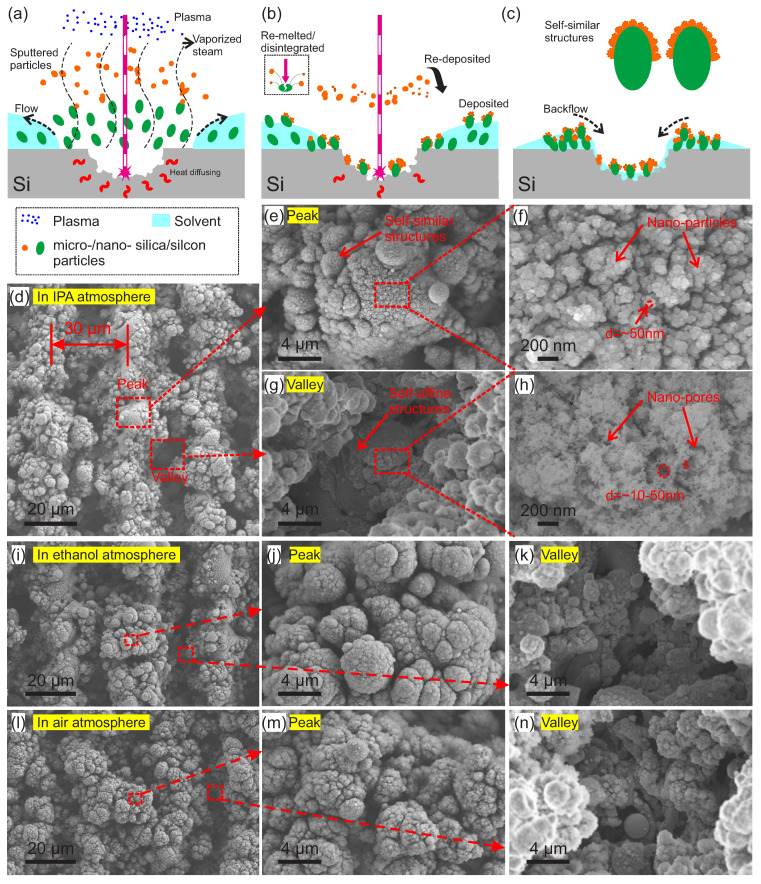
Mechanism of self-similar hierarchical micro–nano structure formation and more detailed micro-views. (**a**–**c**) Schematics of texture forming process on black Si: (**a**) Laser ablating Si surface that led to sputtered particles, vaporized plasma, and plasma; (**b**) re-melted and disintegrated particles under pulsed laser beam and hierarchical structures that were generated due to depositing and re-depositing; (**c**) solvent flowing back and self-similar hierarchical structure stacking. (**d**–**h**) Field scanning electron microscopy (FSEM) images of a black Si sample under *v* = 15 mm/s and *ΔL* = 0.03 mm in the IPA atmosphere: Micro-view of surface topography containing microgrooves (**d**); the magnified image of a “peak” with several broccoli-like structures (**e**); a further amplified micro-view of a broccoli-like structure that is rich in nano-particles (**f**); the magnified image of a “valley” with several broccoli-like structures (**g**); a further amplified micro-view of a broccoli-like structure that is rich in nano-pores and nano-particles (**h**). (**i**–**k**) FSEM images of a black Si sample with the same parameters as the above but in an ethanol atmosphere: Overview in (**i**) and magnified views of a “peak” (**j**) and “valley” (**k**) that contained abundant broccoli-like structures. (**l**–**n**) FSEM images of a black Si sample with the same parameters as the above but in an air atmosphere: Overview in (**i**) and magnified views of a “peak” (**j**) and “valley” (**k**).

As shown in Figure 2a,b, the ns-UV laser introduced a fast pulsed and concentrated energy-dense spot on the silicon surface, subsequently leading to a series of chemical and physical interactions. Firstly, the laser in the ultraviolet spectrum was often accompanied by strong chemical reactivity. Consequently, the silicon crystal on the surface was impacted and destroyed, and parts of the crystal were directly discomposed into meso-/micro-/nano-scale silicon/silica particles and blasted away radially from the spotting zone. At nearly the same time, the accompanying thermal energy was overloaded and diffused into Si bulk or blasted parts from the surface to the interior, which resulted in the melting of micro-/nano-sized silicon/silica particles. Additionally, the laser energy led to an instant solvent (IPA/ethanol) vaporization close to the spotting zone. Subsequently, the disintegrated micro-/nano- particles were carried by the vaporized solvent from the Si surface to the air, where the smaller particles were sputtered a bit further since they were less sensitive to the gravity-led sedimentation. During the break windows of laser beam shooting, the larger particles were preferentially deposited on surface due to their shorter distance from Si surface than the smaller ones. Along with the high frequency pulse repetition, the molten or sputtered larger particles were partially impacted by laser beam and re-melted or even broken down into smaller nanoscale ones that were re-sputtered and redeposited on the surfaces of the larger particles of Si. Such thermal expansion and melting of the Si bulk, along with the disintegration, sputtering, and deposition of particles, were repeated again and again with the continuous shooting of nanosecond laser pulse. Finally, self-similar hierarchical structures with meso-/micro-/nano-scales were formed. In brief, the thermally expanded Si bulk with a sub-melted surface was combined with hierarchically re-deposited mesoscale, microscale, and nanoscale particles, thus leading to the final surface topography.

Based on the above processes, self-similar hierarchical broccoli-like structures were finally formed around laser spotting zone, and they were accompanied with micro-/nano-pores due to Si bulk splashing and solvent evaporation (Figure 2b). In addition, the laser-induced plasma restricted the range of micro-particle splashing with its plasma electric field, which have helped form regular surface textures (Figure 2a). Furthermore, after laser beam scanning, the solvent flowed back and carried hierarchical structures to the “valleys” and “peaks,” which generated regular microgrooves that are abundant in hierarchical structures; see Figure 2c (the corresponding microgrooves are shown in Figure 2d,i). As an extra effect, a plasma vaporization process accompanied the UV lasering. It consequently facilitated the oxidization of surface elements via oxygen doping, therefore contributing to light absorption enhancement via induced impurities. Furthermore, by comparing the samples in the IPA/ethanol bath during lasering, we found that there were three benefits of this process. Firstly, extra heat was partially dissipated via solution evaporation, which prevented overheating and further destroyed the interior part of the silicon wafer; see Figure 2b. Secondly, it was useful for effectively doping elements of carbon and oxygen from the solvent, which may have promoted the light absorption in the infrared spectrum. Thirdly, the backflow of the ambient liquid environment helped re-deposit micro-particles and form regular microgrooves, such as that micro-seen in Figure 2d,i. However, the backflow in the air atmosphere (Figure 2l) did not.

Further, an FSEM was used to examine the morphologies of the obtained Si samples with *v* = 15 mm/s and Δ*L* = 0.03 mm in IPA, ethanol, and air atmospheres; see Figure 2d–n. In Figure 2d,i, there are several parallel microgrooves with period of ~30 µm that corresponded with the interval of adjacent scanning lines. The width of micro-peaks was ~25 µm, which was almost the same as that of our laser facula. The width of micro-valleys was ~5 µm, which was larger than any wavelength we tested in this work. Additionally, a magnified view of the “peaks” and “valleys” can be clearly observed in Figure 2e–h. Numerous self-similar broccoli-like structures on both “peaks” and “valleys” can be seen there. Detailed quantitative measurements indicated that there were nanoparticles with a size of ~50 nm (Figure 2f) and nano-pores with a size of ~10–50 nm (Figure 2h) on broccoli-like “peaks” and “valleys” structures, respectively. These hierarchical micro-/nano-structures helped to increase the diffusive reflection of light, thus increasing the possibility of light reflecting from “peaks” into “valleys,” and the light was finally captured/trapped by micro/nano-pores and micro/nano, broccoli-like, self-similar structures in “valleys.” Consequently, the light absorbance of the treated Si samples was effectively improved. In addition, in Figure 2i–k, one can observe parallel microgrooves and broccoli-like structures on both the “peaks” and “valleys” of the sample fabricated with ethanol. As can be seen, the absence of nano-pores may have reduced the ability to trap light. As shown in Figure 2l–n for the samples treated in air, there were less “valleys” but more “ridges.” A possible reason for this is that the sample was treated in an ambient air environment, and the melted particles sputtered more dispersively without the backflow of the ambient liquid. Thus, less hierarchal self-similar structures were formed, and those that did had a lower fractal dimension (see Figure 1c), two aspects that were not good for capturing light in a broadband spectrum. Above all, these hierarchical structures varied from the nanoscale to the microscale, contributing to high light trapping in the broad spectrum, particularly in a long wavelength range compared to its peers, therefore improving light absorbance [40,61,64].

### 3.3. Parameter Tuning and Surface Characterization

**Figure 3:**
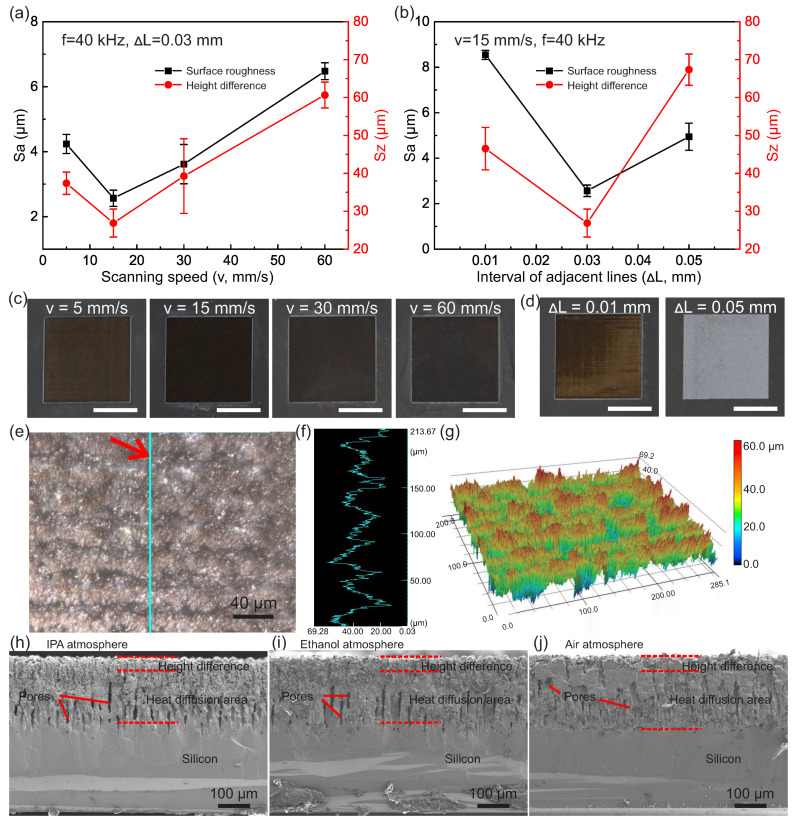
Surface morphology versus UV-laser operating parameters. (**a**,**b**) Surface roughness (Sa) and height difference (Sz) of surface texture of the UV-laser-treated Si with different scanning speeds (5, 15, 30, and 60 mm/s) (**a**) and different intervals of adjacent scanning lines (0.01, 0.03, and 0.05 mm) (**b**). (**c**,**d**) Corresponding optical photos, scale bars indicate 5 mm. (**e**) The magnified planar view of a 285 × 200 µm^2^ area of black Si with *v* = 15 mm/s and Δ*L* = 0.03 mm in an IPA atmosphere. (**f**) The corresponding surface profile of the green line in (**e**). (**g**) The corresponding 3D surface topography observed under the laser scanning confocal microscope. (**h**–**j**) Cross section view of black Si (*v* = 15 mm/s and Δ*L* = 0.03 mm) treated in the IPA (**h**), ethanol (**i**), and air (**j**) atmospheres observed under FSEM.

To technically tune and characterize the surface morphology [65], we adjusted the scanning speed and space between the adjacent scanning lines of the UV-laser; see Figure 3. In our tests, after increasing the scanning speed from 5 to 60 mm/s, the surface roughness of treated surface firstly decreased to the minimum (~2.57 µm) and then increased nearly linearly; see Figure 3a. Meanwhile, the corresponding height difference also showed a similar tendency, and the minimum was ~26.89 µm. The result indicated that the treated surface exhibited a rougher texture when the scanning speed was less or more than 15 mm/s, and the height difference was affected in the same way. A similar tendency was observed when we adjusted the space between scanning lines (from 0.01 to 0.03 and 0.05 mm) while the other parameters were kept constant; see Figure 3b. Furthermore, corresponding optical photos are shown in Figure 3c,d. Apparently, the sample with parameters of *v* = 15 mm/s and *ΔL* = 0.03 mm was the blackest and had the highest light absorbance efficiency, while the other samples showed non-uniform color or lighter blackness.

In addition to the planar optical photos, topography quantification was also conducted to explore the surface (Figure 3e–g). In the planar view (Figure 3e), the lighter areas refer to “valleys” and the darker areas refer to “peaks,” which indicates an alternative arrangement of the hierarchical structures. Additionally, such similar arrangements of “valleys” and “peaks” can be observed in a 3D plotted view (Figure 3g). Specifically, the cross profile in Figure 3f shows a robust and uniform height difference of ~30 µm. Furthermore, more detailed micro-views of cross section treated in IPA, ethanol, and air atmospheres are shown in Figure 3h–j. All samples exhibited an effectively roughed layer from direct lasering (~30 µm height difference), a sub-melted layer due to heat diffusion (~150–200 µm), and a bottom layer of silicon bulk.

In addition, we investigated the surface morphology of cPDMS under various operation parameters (Figure S3). In IPA and air, the surface roughness and height difference increased with interval of adjacent lines but decreased with scanning speed. According to the 3D profiles, compared to that treated in the air, a sharper and higher aspect ratio topography was obtained in IPA. This observation could be useful for surface performance enhancements, such as radical traps, solvent absorption, and corresponding swelling

**Figure 4:**
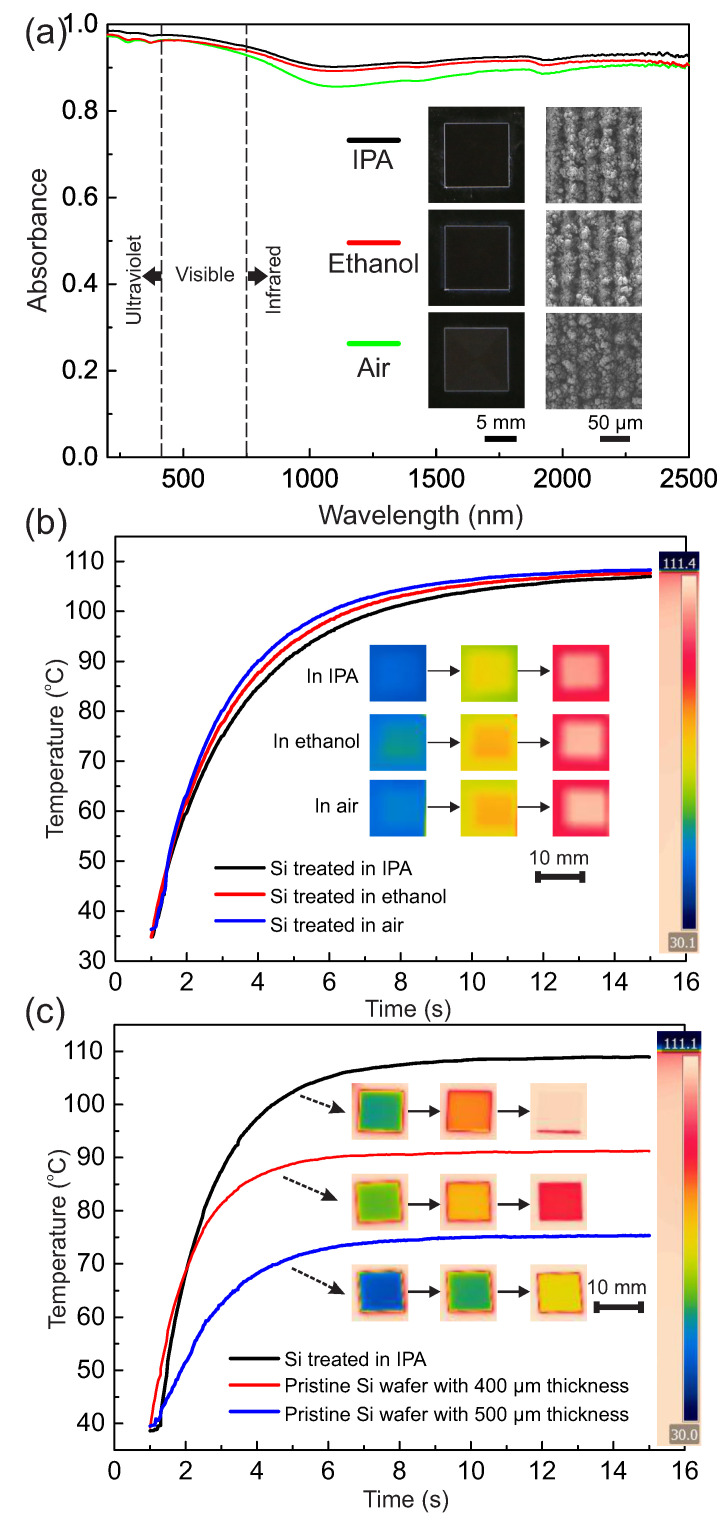
Light absorbance and heat transfer features of the treated Si samples. (**a**) Light absorbance of three Si samples in the 200–2500 nm range. All these three samples were treated with the same parameters of *v* = 15 mm/s and Δ*L* = 0.03 mm but in different atmospheres of IPA (the black curve), ethanol (the red curve), and air (the green curve). (**b**,**c**) Temperature measurement during the heating process: (**b**) comparison curves of the three Si samples treated in IPA, ethanol, and air; (**c**) comparison curves of the treated Si samples with an original thickness of 500 µm, the pristine Si wafer with a thickness of 400 µm, and the pristine Si wafer with a thickness of 500 µm. All samples had an area of 10 × 10 mm^2^.

Regarding light trap, we measured the absorbance of three Si samples treated with *v* = 15 mm/s and Δ*L* = 0.03 mm in IPA (black curve), ethanol (red curve), and air (green curve) atmospheres in the spectral wavelength of 200–2500 nm (Figure 4a). For the Si treated in the IPA bath in the 200–400 nm spectral range (ultraviolet), the absorbance kept over 97.5%; in the visible range (400–750 nm), the absorbance showed a slight decrease but still stayed over 94.9%; and in the infrared range (750–2500 nm), the absorbance remained up to 90%. Meanwhile, in the Si treated in ethanol and air atmospheres, the absorbance (>88% and >85%, respectively) was a little less than that of treated in the IPA bath. Insets of the figures show the corresponding macro-/micro-view of these three samples. In addition, according to previous studies [61,66], the doping of oxygen may have formed Si–O bonds and improve the impurity band absorbance in the infrared range (Figure S4). Additionally, such a multiscale (from tens of nanometers to tens of micrometers) hierarchical structure guaranteed that there were always structures smaller than any wavelengths in our test that could interact with the incident light. Additionally, the self-similar configuration also helped to trap and absorb light by increasing numbers of light reflecting in the structures. More light absorbance curves from the samples under different laser operation situations can be found in Figure S5. Those results further revealed a robust high and broadband light absorbance performance with such self-similar hierarchical micro–nano structures due to the high factual dimension and broad feature size coverage (from tens of nanometer to tens of microns).

Moreover, we studied the heat transfer process of three Si samples with the operational parameters as described above (Figure 4b), and we compared Si samples treated in IPA with two pristine Si wafers with thicknesses of 400 and 500 µm (Figure 4c). The insets of the figure show the surface temperature changes of the samples along with the heating process on a hot plate heated to 110 °C. Figure 4b reveals that UV-lasered samples were heated up to 105 °C within 15 s at similar heating rates. Various broccoli-like self-similar structures on the surfaces further increased the specific surface area of the hierarchical structures, thus improving the heat transfer efficiency. Additionally, the laser-treated sample shown in Figure 4c was heated up to 110 °C, which was much higher than that of the pristine Si wafers with thicknesses of 400 µm (90 °C) and 500 µm (75 °C). This indicated that broccoli-like self-similar structures immensely contributed to the heat transfer. More curves of various Si samples can be found in Figure S6.

### 3.4. Functional Surfaces

**Figure 5:**
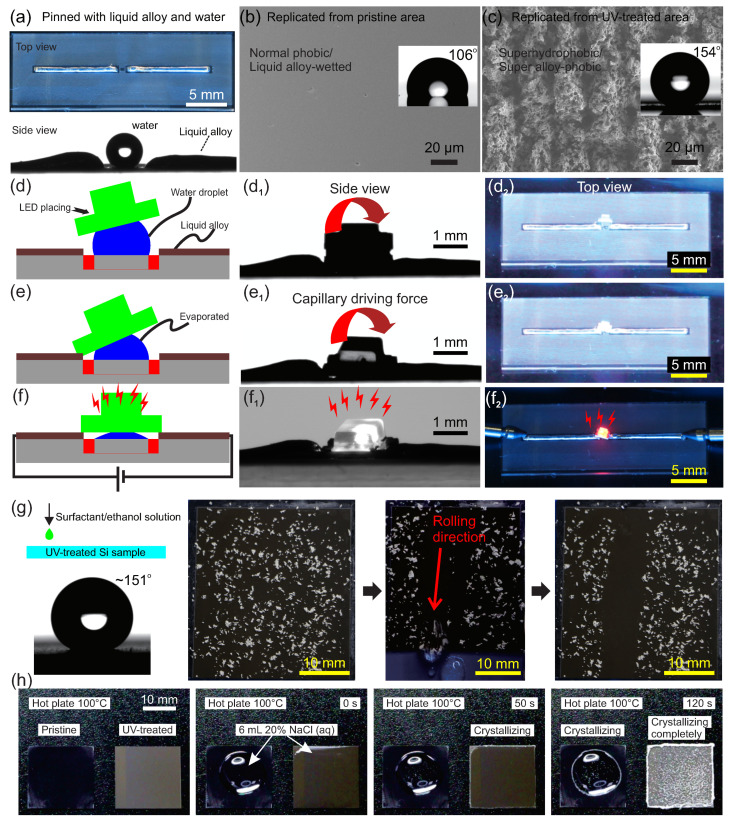
Demonstrations of Si with self-similar hierarchical micro–nano structures. (**a**–**f**) LED self-assembly with a replicated PDMS substrate from the treated Si master. (**a**) Top view and side view of the replicated PDMS substrate with a hydrophobic/liquid alloy wetted and superhydrophobic/super liquid alloy-phobic areas, which were pinned with liquid alloy lines and water droplets (side view). (**b**,**c**) FSEM views of areas replicated from pristine Si (**b**) and UV-treated Si (**c**). Insets show a static contact angle of a 2 µL water droplet on these two surfaces. (**d**–**f**) Schematic process of LED self-assembly via a capillary driving force along the evaporated water droplet. (**d_1_**–**f_1_**) Side view of self-assembling process. (**d_2_**–**f_2_**) Top view of the self-assembling process. (**g**) A self-cleaning demonstration via a slightly tiled (5°) and perfluorooctyl trichlorosilane-treated Si sample. The dotted white points are chalk dust. (**h**) A heated salt crystallization demonstration on the Si surface with self-similar structures and a pristine one.

After selectively UV-lasering, the patterned Si could be used as a replica master for patterning PDMS substrates (Figure S7). In order to ease the peeling off of the PDMS from the Si master, a larger space of *ΔL* = 0.06 mm was selected with *v* = 15 mm/s in air. The replicated PDMS was patterned with a rough zone (the frosted) and a flat zone (the transparent), which were superhydrophobic/super-liquid alloy-phobic and hydrophobic/liquid alloy-wetted, respectively (Figure S8a). Such a surface induced the anisotropic wetting behavior in Figure 5a and can be used for the self-assembly of an electronic component (e.g., LED) connected to a patterned liquid alloy circuit via the capillary effect [67,68,69]. This selective micro–nano-roughed surface helped to stabilize water droplet and liquid alloy lines on water/alloy-wetted areas while repelling them on the phobic areas (side view). FSEM views in Figure 5b,c and Figure S8b–f indicate that the surface morphology of the PDMS was faithfully replicated from the patterned Si, as in the abundant micro/nanoscale pores on superhydrophobic areas. After selectively pinning liquid alloy lines [70] and water droplets in the middle pristine water/alloy-wetted area, an LED was placed on a water droplet (Figure 5d–d_2_). Due to the gravity, the LED slid down and contacted the substrate (or liquid alloy) but was still pinned on the surface of the water droplet. Along with water evaporation, the LED was aligned and placed to contact the liquid alloy lines (Figure 5e–e_2_) due to capillary force. Figure 5f–f_2_ shows the final state of self-assembled LED–liquid alloy circuit. As reveled by the captured images, such a connection process could be clearly observed in our experiment. As shown in Figure 5d_2_, the LED was not connected with the liquid alloy in the beginning. By enhancing evaporating temperature, the assembly could be accelerated to meet the demand; see Figure 5f_2_. The results indicated that such selectively patterned micro–nano multiscale structures can be utilized for the hybrid integration of rigid components and liquid alloy lines, which could be useful for the future mass production of soft electronics.

As expected, our lasered hierarchical structures on the Si surfaces exhibited super hydrophilic behavior. After being dripped, the water droplet initially spread out with a contact angle of <10° and was subsequently absorbed in the treated Si surface with a contact angle of ~0° (Figure S9 and Video S1). Furthermore, the lasered Si sample was easy to be tuned to superhydrophobic (with a contact angle of ~151°) via 1H, 1H, 2H, and 2H-perfluorooctyl trichlorosilane/ethanol solution treatments, thus exhibiting an excellent self-cleaning feature. As revealed in Figure 5g, the chalk dust could be easily wiped away while the water droplet rolled on the surface by slightly tilting the surface to a small angle (5°). Due to its high effective surface area in such a hierarchical structure, the sample could also be used for the fast crystallization of NaCl via water evaporation; see Figure 5h. Such behavior may impact light-heat-induced sea water purification or heat transferring and dissipation for high density integrated electronics.

**Figure 6:**
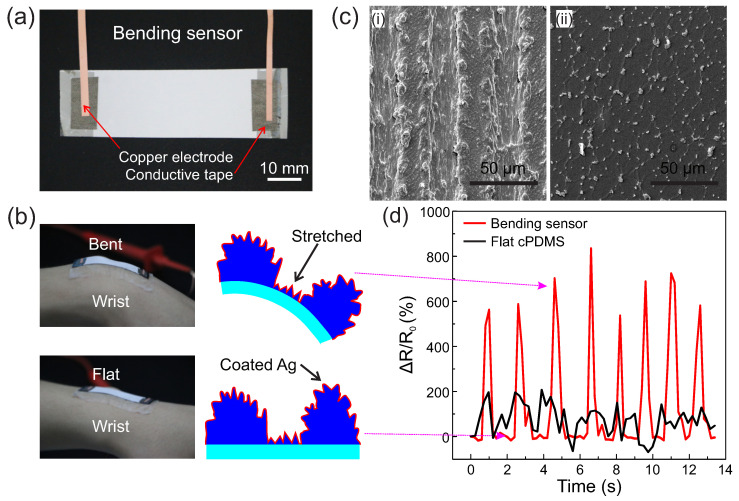
Demonstration of bending sensor with broccoli-like hierarchical micro–nano structures. (**a**) A cPDMS bending sensor with hierarchical micro–nano structures coated with a 100 nm thick layer of Ag. (**b**) The bending sensor and the corresponding micro-schematics. (**c**) FSEM views of the bending sensor (**i**) compared with the flat cPDMS (**ii**) after a bending test. (**d**) The relative resistance rate of the bending sensor (the red curve) laminated on the wrist under dynamic motions compared with that of the flat cPDMS (the black curve).

Furthermore, to exploit more potentials of the hierarchical micro–nano structures on diverse materials, we fabricated a bending sensor with the surface of this multiscale structures that was coated with an extra thin (~100 nm) layer of Ag; see Figure 6a. After being laminated onto a volunteer’s wrist, the bending sensor deformed by following the wrist motions; see Figure 6b. As observed in Figure 6c, the surface of the bending sensor comprised periodical microgrooves, which resulted in larger strains in the “valleys” due to a localized stiff gradient caused by a height difference in the cross-scale micro–nano structures. In contrast, the compared flat cPDMS showed no obvious microgrooves. Thus, the relative resistance change rate could have been up to 800%, which was much higher than that of the flat cPDMS; see Figure 6d. The result showed that the bending sensor had a high application potential for sensitive soft/wearable sensors.

### 3.5. Brief Discussions and Perspectives

Figure S10 and Table S1 summarize our fabrication approach, along with others, for the application of light absorbance, including employed instruments, fabrication atmospheres, and solutions. Generally, our major equipment was a cost-effective UV-laser marker that led to low equipment and facility investments. Additionally, our process is quite simple in practical operations when compared to other examples. In particular, our black Si showed a high and stable light absorbance in a broad wavelength range, especially in the infrared spectra. Though we only demonstrated high light absorbance, super hydrophilicity, and highly efficient heat transfer of such broccoli-like, self-similar, hierarchical micro–nano structures on silicon in this work, we believe the potential of such structures is far from thoroughly exploited. Furthermore, by doping with carbon, our approach can been extended to soft materials such as silicone. Actually, this kind of doping works well for many other soft materials, such as polyvinyl alcohol (PVA), EcoFlex, and polyurethane (PU). Our method is very able to be a versatile technique with proper, minor modifications.

As is known, self-similar structures such as fractal antennas, metamaterials, and ultra-capacitors are widely used in many fields. Considering the natural similarity behaviors in many diffusion-based processes, we believe such self-similar hierarchical micro–nano structures can be further extended to many other similar scenarios, e.g., electrodes in batteries, triboelectric generators, catalysis carriers for gas/liquid–solid reactions, and selective cell patterning/tissue engineering. Though we strived to demonstrate some applications to exploit its advantages, more investigations are necessary in the future. They may lead to a new path to an undiscovered world where our fabrication technique may serve as a useful tool.

## 4. Conclusions

In conclusion, we established an elegant approach to artificially make self-similar hierarchical micro–nano structures on Si via solvent-assisted UV-lasering. In practical operation, the surface morphology can be tuned by adjusting relevant laser operational parameters and ambient solvents. Through micro observation, abundant broccoli-like structures on silicon were observed in both “peaks” and “valleys,” which were composed of nano-particles and nano-pores with sizes ranging from several to hundreds of nanometers, and such unique, hierarchical, multiscale structures enhance the ability of light trapping in an ultra-broad spectral range. Additionally, this technique can further be utilized for the self-assembly of soft circuits, as a self-cleaning surface, or a highly efficient heat transfer surface. Furthermore, with minor modifications, this technique can be extended to soft materials such as PDMS. Since the potential of self-similar hierarchical micro–nano structures is far from thoroughly exploited, our way may provide an excellent tool to explore unique features of such structures and to further develop diverse exciting applications.

## Supplementary Materials

The following are available online at https://www.mdpi.com/2072-666X/11/7/682/s1. Figure S1: Schematic fabrication process with solvent-assisted UV-lasering; Figure S2: Various surfaces with self-similar structures; Figure S3: Surface morphology of cPDMS; Figure S4: EDX map of black Si samples; Figure S5: Light absorbance curves of black Si samples with different parameters; Figure S6: Heating features of various samples; Figure S7: Detailed fabrication process of soft substrate (PDMS) with water/liquid alloy amphiphilic areas; Figure S8: Micro-views of Si master and replicated PDMS substrates; Figure S9: Superhydrophilicity of Si with self-similar structures; Figure S10: Radar plot of comparison our approach with others; Video 1: Superhydrophilicity of black silicon samples.
