# Supplementary material for: Facile Fabrication of Self-Similar Hierarchical Micro-Nano Structures for Multifunctional Surfaces via Solvent-Assisted UV-Lasering
Source: Micromachines (Basel). 2020 Jul 14;11(7):682. doi: 10.3390/mi11070682 (PMC7407878; doi:10.3390/mi11070682)
Supplement: Supplementary file 1 [file micromachines-11-00682-s001.zip › micromachines-847248 Supplementary/micromachines-847248 supplementary layout.pdf]

micromachines

Supplementary Materials: Facile Fabrication of Self-Similar Hierarchical Micro-Nano Structures for Multifunctional Surfaces via a Solvent Assisted UV-Lasing

Shuo Zhang [1], Qin Jiang [1], Yi Xu [1], Chuan Fei Guo [2] and Zhigang Wu [1,3,*]

[1] State Key Laboratory of Digital Manufacturing Equipment and Technology, School of Mechanical Science and Engineering, Huazhong University of Science and Technology, Wuhan 430074, China; shuo_zhang@hust.edu.cn (S.Z.); jiangqin@hust.edu.cn (Q.J.); xuyi_sr@hust.edu.cn (Y.X.)
[2] Department of Materials Science and Engineering, Southern University of Science and Technology, Shenzhen 518055, China; guocf@sustech.edu.cn (C.F.G.)
[3] School of Optical and Electronic Information, Huazhong University of Science and Technology, Wuhan 430074, China
* Correspondence: zgwu@hust.edu.cn

Received: 10 June 2020; Accepted: 8 July 2020; Published: date

Supplementary Method 1:

A different box-counting (DBC) is used to compute the Fractal Dimension (FD) of texture images [1, 2]. The basic equation of FD is given by

$$D = \frac{log(N_r)}{log(1/r)} \quad (1)$$

The processing steps are as follows:

1. Image preprocessing: Convert to a grayscale image if the image is a color image.
2. Image segmentation: Divide the image with the pixel M×M into a grid of s×s, each grid size is r. r = s/M.
3. In each s×s block, there is a box column s×s×s'. If the maximum gray value is g, usually 255, g/s' = m/s.
4. Calculating the maximum gray value and the minimum gray value of each block, which fall into boxes l and k respectively, then

   $$n_r = l - k + 1 \quad (2)$$

5. Taking contributions from all grids, we have

   $$N_r = \sum_{i,j} n_r(i,j) \quad (3)$$

6. Then using (1), we can estimate D, the fractal dimension, from the least square linear fit of $\log(N_r)$ against $\log(1/r)$.

Matlab (version 2018a) is used for programming calculations. The code is shown as follows:

```
function fd = box_frac_dem(P);

% Example:

P=double(imread('***.png'));

if size(P,1)~=size(P,2);

    error('The size of X must NxN.');

end

B=size(P,1);
```

```
if mod(log2(B),1)>0;
    error('The size of X must 2^n');
end
t=log2(B);
s=2.^(1:t); % s = 2,4,8,16,32,64,128,256 Çó2µÄÃÝ
Nr=zeros(1,t); % nr = 0         0         0         0         0         0         0         0
for k=1:t;
    d=s(k);
    h=256/d; % Let B instead of 256 for the size [2^nX2^n]
    for m=1:h;
        for n=1:h;
            A=P(d*(m-1)+[1:d],d*(n-1)+[1:d]);
            mn=min(A(1:end));
            mx=max(A(1:end));
            nr=fix(mx/d)-fix(mn/d)+1;
            Nr(k)=Nr(k)+nr;
        end
    end
end
r=B./s;
p=polyfit(log10(r),log10(Nr),1); %Least squares
fd=p(1);
```

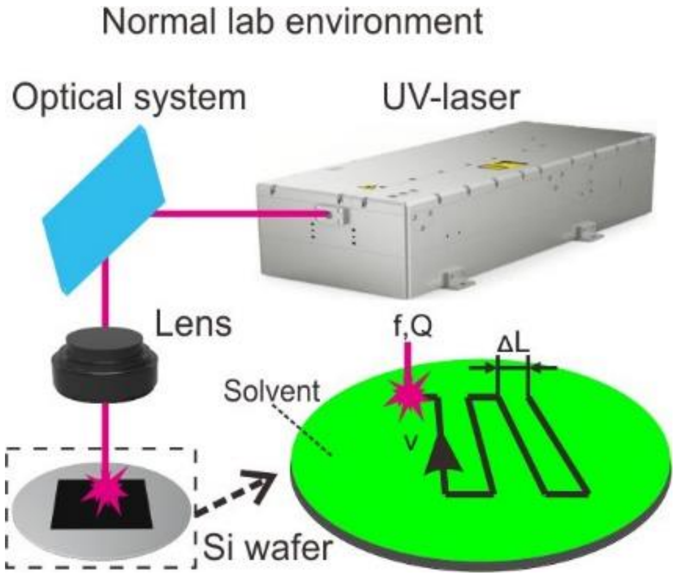

Figure S1. Schematic fabrication process of a solvent assisted UV lasing in an ordinary laboratory environment, in which pulse repetition frequency (*f*) and pulse width (*Q*) of the UV laser are fixed at 40 kHz and 1 μs for Si and 50 kHz and 0.1 μs for cPDMS in this work, respectively, meanwhile the scanning speed (*v*) and internal of adjacent scanning lines (Δ*L*) are tunable. The energy per pulse and duty cycle are 5 μJ and 0.25, respectively.

.

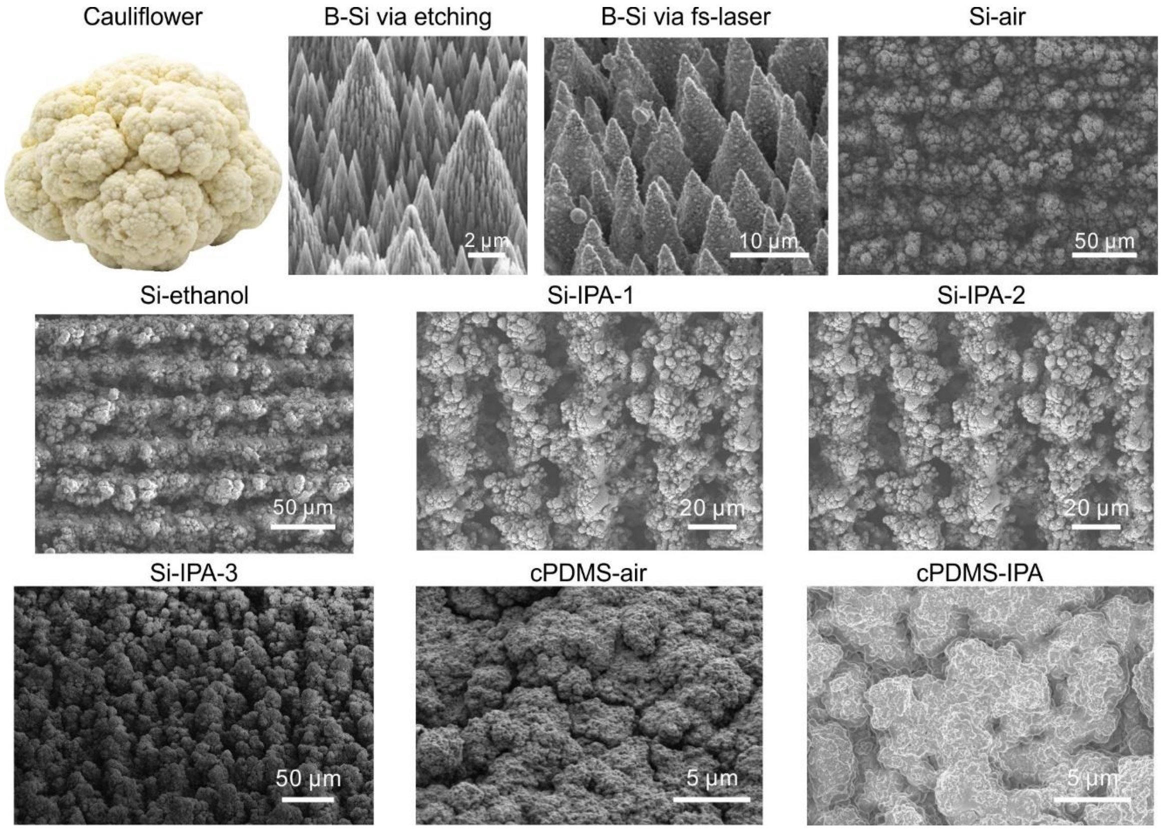

Figure S2. Various surfaces with self-similar structures for fractal dimension calculation in Fig. 1c (from left to right and from top to bottom): cauliflower, black Si sample via etching [66], black Si sample via femtosecond laser ablation [3], Si treated in air/ethanol/IPA via UV-laser, carbon-PDMS (cPDMS) treated in air/IPA via UV-laser.

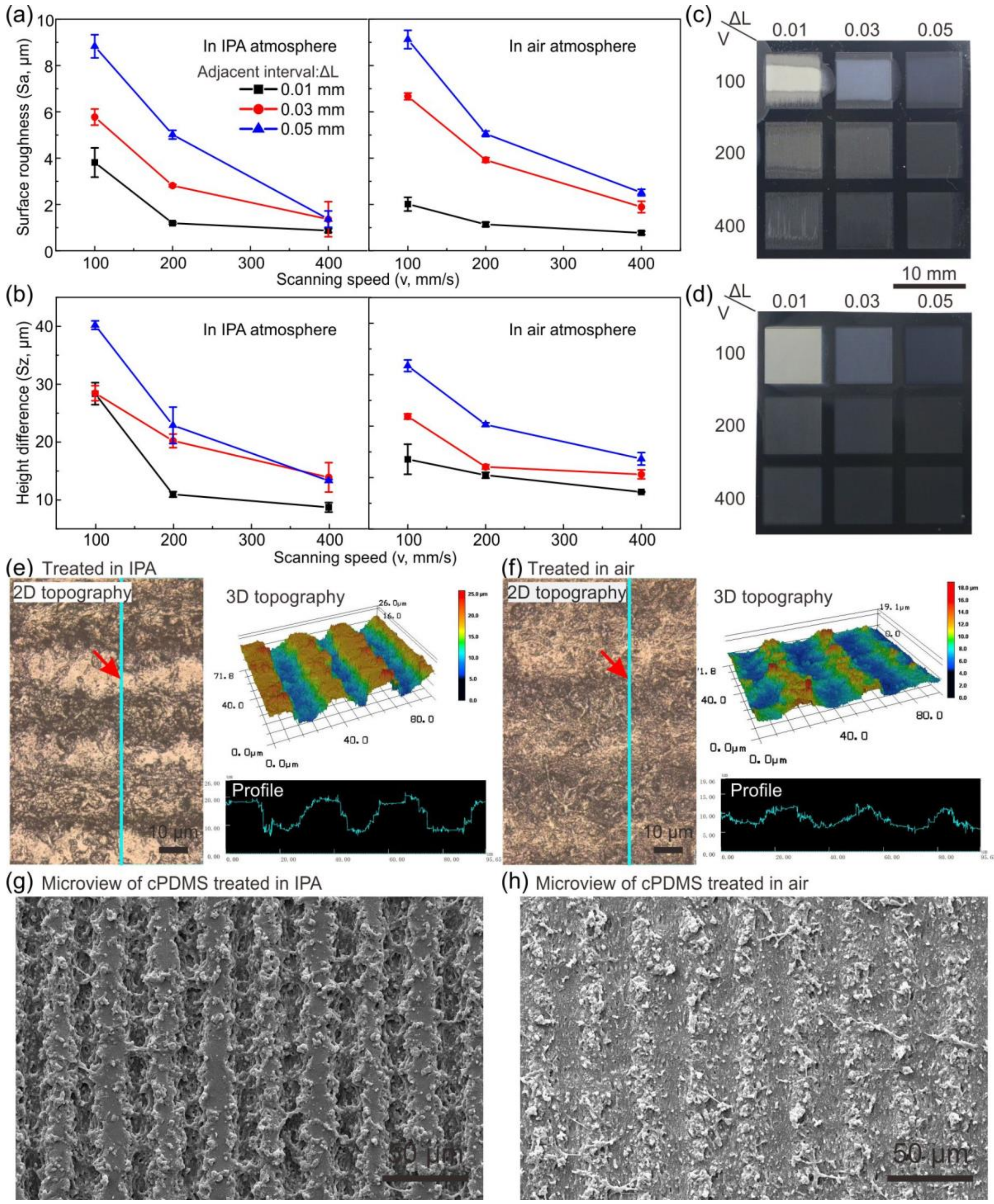

Figure S3. Surface morphology of cPDMS (**a**,**b**) Surface roughness (Sa) and height difference (Sz) of surface texture of the UV-laser treated cPDMS samples with different scanning speeds (100, 200 and 400 mm/s) and different intervals of adjacent scanning lines (0.01, 0.03 and 0.05 mm) in IPA and air environment. (**c**,**d**) Visual photos of the corresponding samples. (**e**–**f**) The planar view, 3D view and corresponding surface profile of treated cPDMS samples under v = 400 mm/s and ΔL = 0.03 mm observed by a laser scanning confocal microscope. (**g**–**h**) The corresponded FSEM micro-views.

When conducting the same process on cPDMS surface, due to the doped carbon black, the cPDMS matrix can absorb the energy of ultraviolet laser, which resulting in the rubber matrix being ablated, micro-/nano- particles sputtered and even vaporized. Under impact by pulse laser, the rubber matrix is anisotropically roughed. The accompanying thermal energy of laser is overloaded and diffused into silicone bulk or blasted parts from the surface to the interior, which results in melting micro-/nano-sized particles and bumps as well. Combing with particles depositing in

solvent, the multiscale structures are generated. In addition, the employed solvent helps form the regular microgrooves in case of the high laser energy inducing much more heat affect zone._

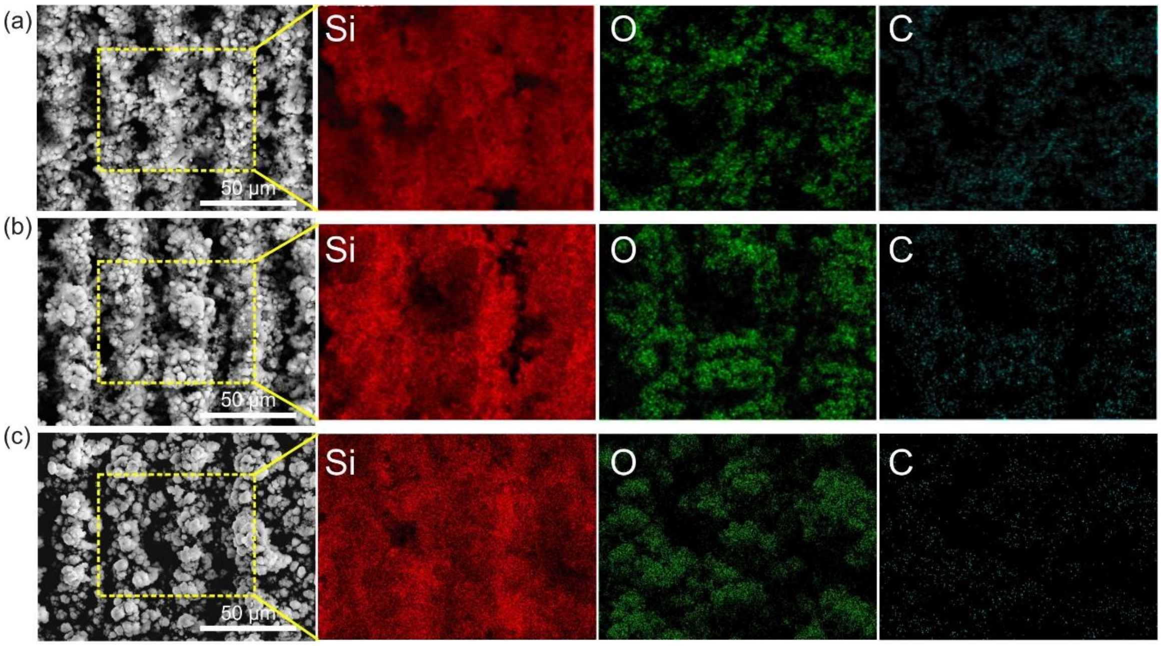

Figure S4. Elemental map of an area of 300 × 300 μm^2 of black Si sample treated in IPA atmosphere **a**), ethanol bath **b**) and air atmosphere **c**). The red refers to silicon, the green refers to oxygen and the blue refers to carbon.

.

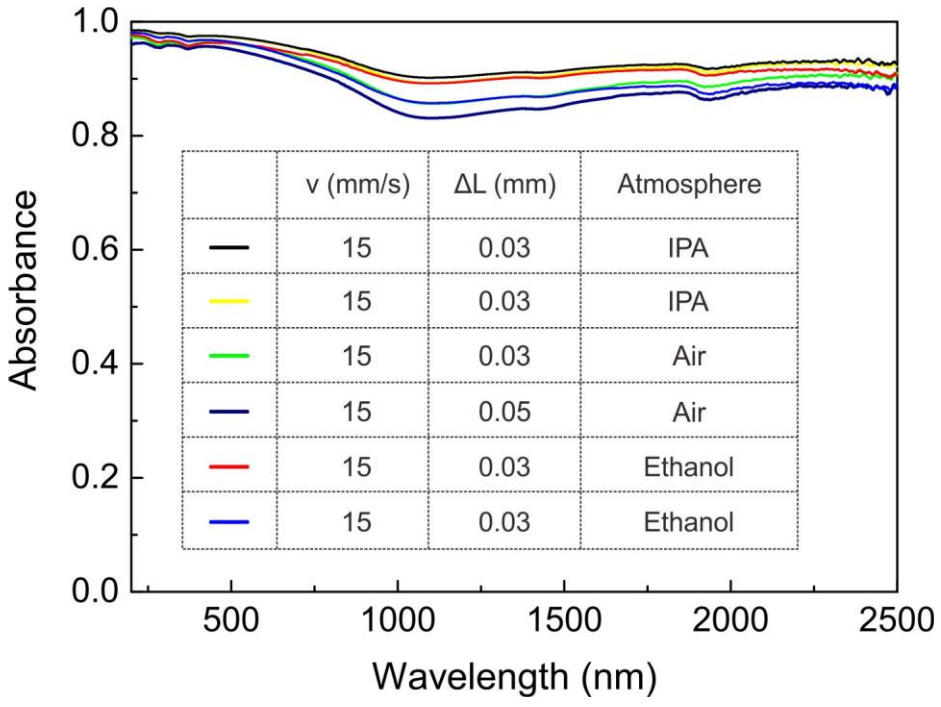

Figure S5. Light absorbance of several lased Si samples in various situations.

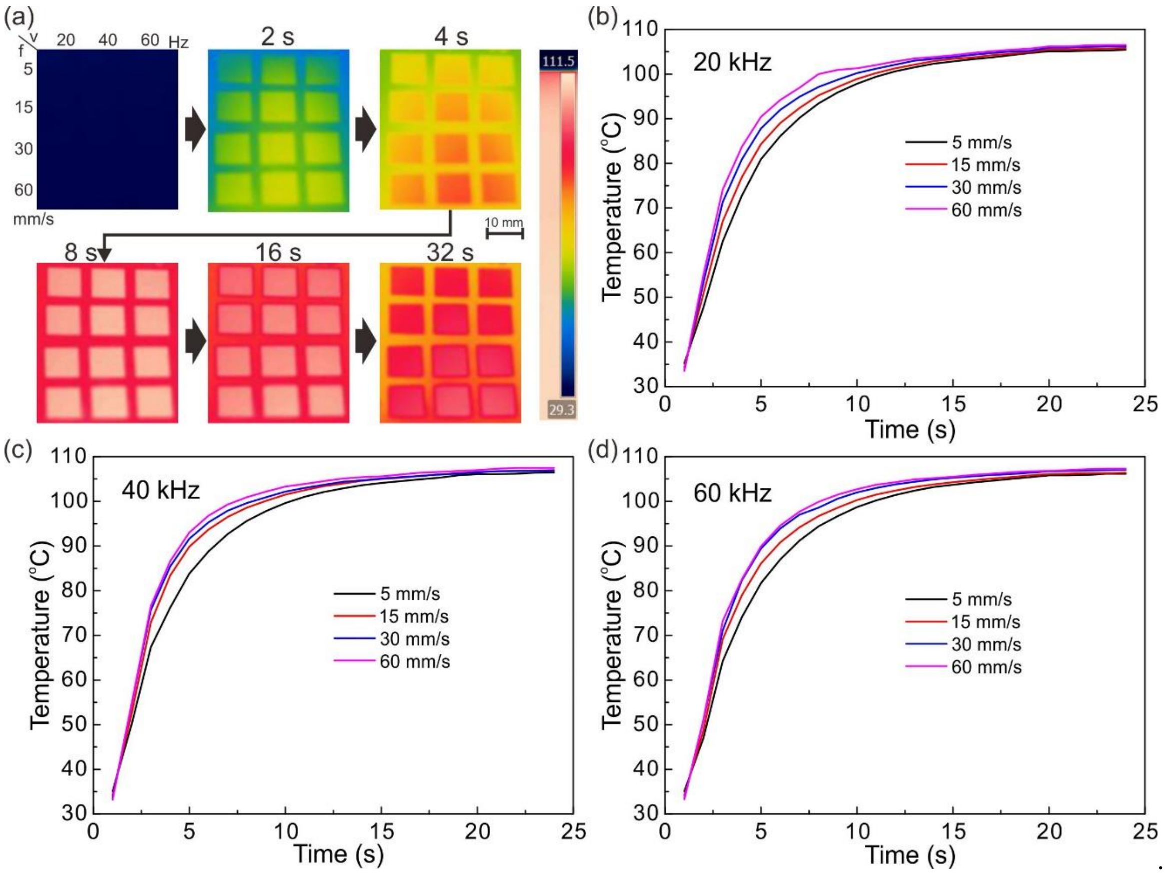

Figure S6. Temperature visualization of the heating process with several lased Si samples under 110 °C. (**a**) The infrared temperature photographs of the samples in heating process. (**b**–**d**) Temperature curves of samples treated with parameters of different scanning speeds (*v*) and different frequency, *f* = 20 kHz **b**), *f* = 40 kHz **c**) and *f* = 60 kHz **d**).

.

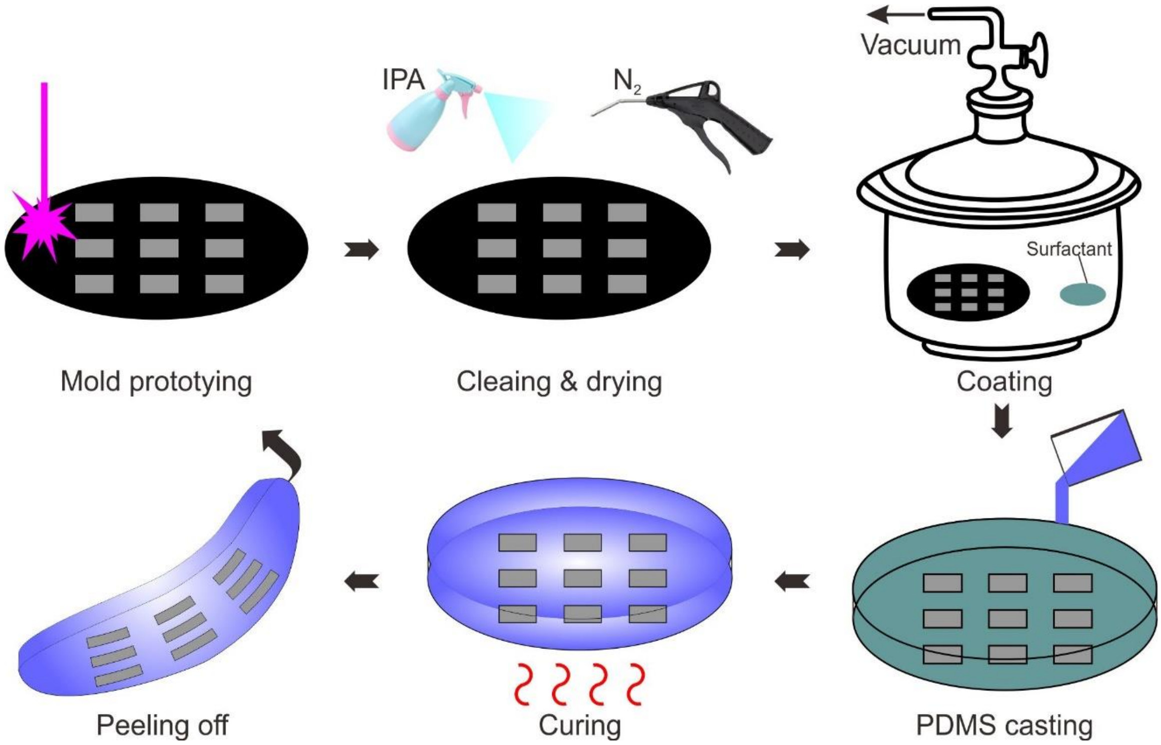

Figure S7. Detailed fabrication process of soft substrate (PDMS) with water/liquid alloy amphiphilic areas with a selectively UV-lased Si master for self-assembling LED via a capillary force.

.

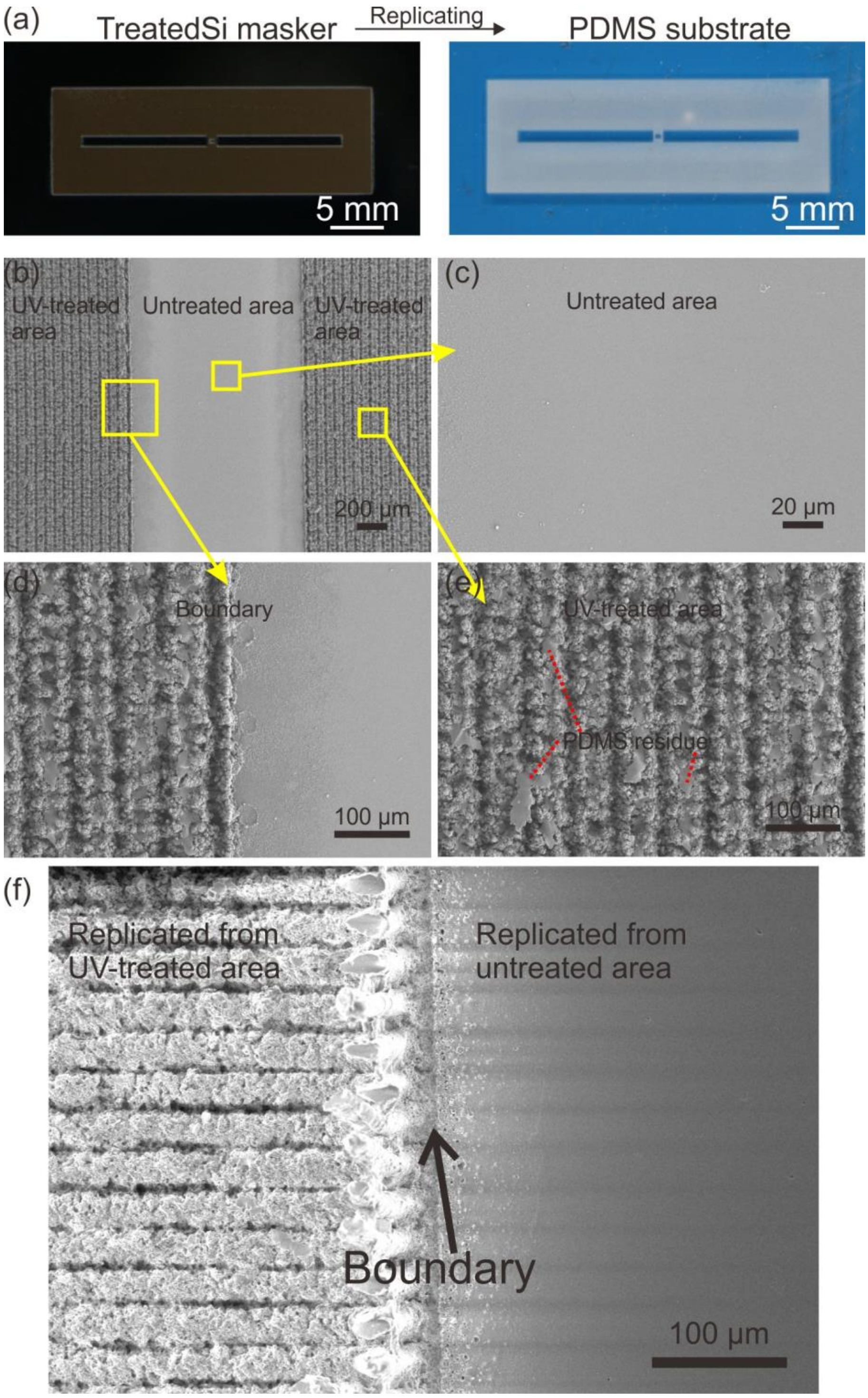

Figure S8. Micro-views of Si master and replicated PDMS. (**a**) The patterned Si master treated with v = 15 mm/s and ΔL = 0.06 mm in air atmosphere, and the replicated PDMS with superhydrophobic/super liquid alloy-phobic areas (the frosted areas) and normal hydrophobic/liquid alloy-wetted areas (the transparent areas). (**b**–**e**) FSEM view of UV-patterned Si master for PDMS replicating. (**f**) FSEM view of the boundary of the PDMS substrate replicated from the lased Si master.

.

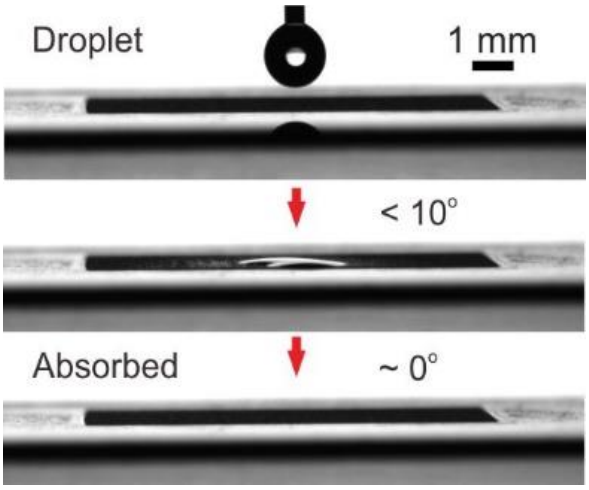

Figure S9. Superhydrophilicity of Si with self-similar structures. A droplet of water-based solution dipped on the treated Si surface.

.

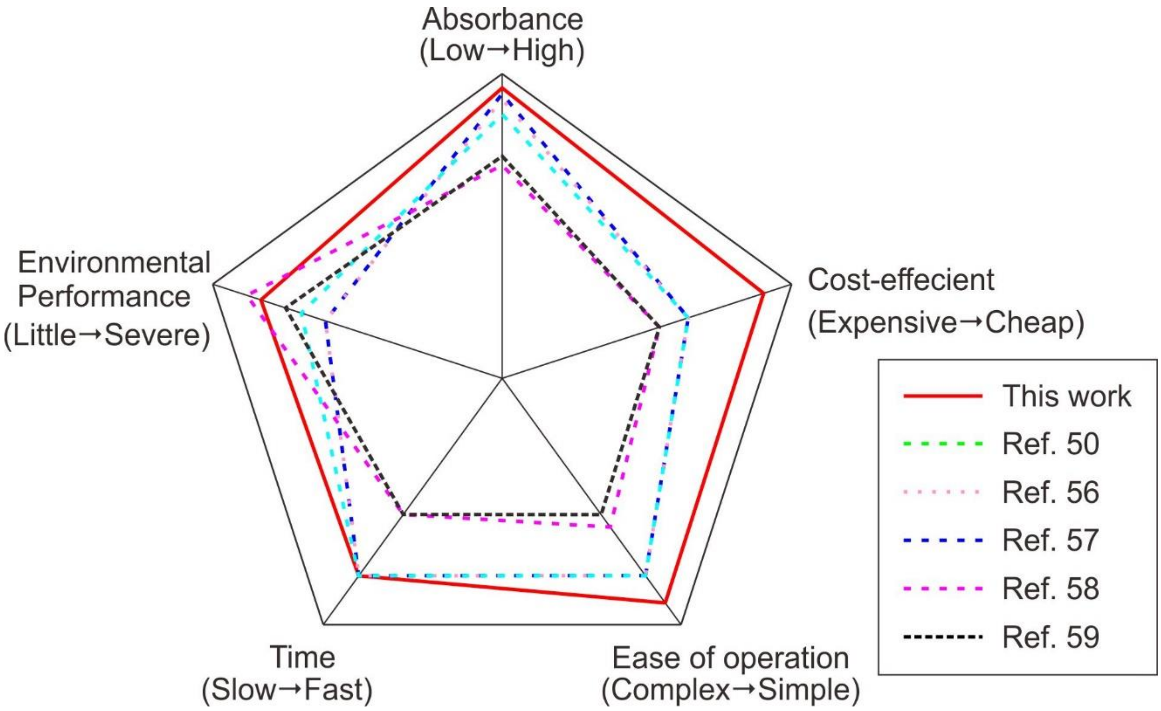

Figure S10. Radar plot of properties of this approach and others.

.

Table S1. Comparison of this method with others.

Method	Atmosphere/Solution	Absorbance/Reflection
Femtosecond laser[57]	SF_6	Absorbance: ~97% in 250–1000 nm; ~90% in 1000–2500 nm
Femtosecond laser[56]	H_2S	Absorbance: ~95% in 250–1000 nm; ~90% in 1500–2500 nm
Femtosecond laser[50]	2 wt% NaOH	Reflection: minimum 5% in 400–750 nm
Metal-assisted chemical etching[58]	Ag layer and N_2	Absorbance: ~95% in 300–700 nm; decreased to ~55% in 700–1100 nm
Metal-assisted chemical etching[46]	Ag	Absorbance: ~92% from 400–1000 nm; ~70% from 1500–2500 nm
Wet etching[59]	KOH and Au-induced HF/H_2O_2 solution	Absorbance: ~90% in 250–1000 nm
This method (UV-laser)	Normal lab environment and IPA	Absorbance: >97.5% in 200–400 nm; >94.9% in 400–750 nm; >90% in 750–2500 nm

micromachines

MDPI

Supplementary References

[1] N. Sarkar, B. B. Chaudhuri. *IEEE Trans. on Syst.*, **1994**, *24*, 115.

[2] J. D. Souza , S. P. Rostirolla. *Comput. Geosci.*, **2011**, *37*, 241.

[3] B. Franta, D. Pastor, H. H. Gandhi, P. H. Rekemeyer, S. Gradecak, M. J. Aziz, E. Mazur. *J. Appl. Phys.*, **2015**, *118*, 225303.
